# Dynamics of Water Quality and Microbial Communities in the Middle Route of the South-to-North Water Diversion Project: Characterization and Driving Mechanisms

**DOI:** 10.3390/microorganisms13081895

**Published:** 2025-08-14

**Authors:** Xinyong Liu, Zhibing Chang, Li Liu, Juechun Li, Jing Gao, Yingcai Wang, Yuming Su, Yuxin Hu, Yu Peng

**Affiliations:** 1 China South-to-North Water Diversion Middle Route Corporation Ltd., Beijing 100038, China; langzijian-007@163.com (X.L.); changzhibing@nsbd.cn (Z.C.); ljc18502410571@163.com (J.L.); 2 Hubei Provincial Key Laboratory for Basin Ecology Intelligent Monitoring-Prediction and Protection, Wuhan 430010, China; 22209010115@stu.wit.edu.cn (J.G.); wangyingcai@cjjg.mee.gov.cn (Y.W.); suyuming@whu.edu.cn (Y.S.); 3 North China University of Water Resources and Electric Power, Zhengzhou 450045, China; liuli2019@ncwu.edu.cn; 4 Changjiang Basin Ecology and Environment Monitoring and Scientific Research Center, Changjiang Basin Ecology and Environment Administration, Ministry of Ecology and Environment, Wuhan 430010, China

**Keywords:** South-to-North Water Diversion Project, microbial communities, water quality, seasonal succession

## Abstract

Microbial communities, as critical functional components of riverine ecosystems, play a pivotal role in biogeochemical cycles and water quality regulation. The South-to-North Water Diversion Middle Route Project (SNWD-MRP) is a major cross-basin water transfer initiative, and bacteria are essential for the stability of water quality in the project. This study employed environmental DNA (eDNA) metabarcoding targeting the 16S rRNA gene to investigate spatiotemporal variations in water quality and bacterial communities along the SNWD-MRP during summer and winter. Integrated analyses, including redundancy analysis (RDA), Mantel tests, and ecological network modeling, were applied to unravel the driving mechanisms of microbial succession. The water quality along the SNWD-MRP is generally classified as Grade I, with significant seasonal variations in water quality parameters and microbial community composition. In the summer, higher temperatures lead to an increased abundance of cyanobacteria. In contrast, during the winter, lower water temperatures and higher dissolved oxygen levels result in the dominance of *Pseudomonas* and *Bacillota* species. RDA identified the permanganate index as the primary driver of microbial composition across seasons, with total phosphorus and total nitrogen having a greater influence in winter. Mantel tests highlighted significant correlations between Cyanobacteria and total phosphorus during winter. Ecological network analysis revealed that the complexity and connectivity of the winter network increased, likely due to suitable nutrient levels rendering the microbial network more complex and stable. These findings underscore the synergistic effects of temperature and nutrient availability on microbial succession, providing actionable insights for optimizing water quality management and ecological stability in large-scale water diversion systems.

## 1. Introduction

Microorganisms, as core functional components of river ecosystems, govern biogeochemical cycles (e.g., carbon, nitrogen, and phosphorus) and energy flows in aquatic systems [1,2,3]. Their community composition and diversity not only serve as critical bioindicators of water quality health [4,5] but also profoundly influence ecological security by regulating pollutant degradation efficiency and algal proliferation dynamics [6,7,8]. Shifts in microbial communities may directly impact water quality, triggering odor issues or health risks [9,10,11]. Seasonal environmental fluctuations—such as changes in water temperature, dissolved oxygen, and nutrient levels—significantly alter microbial metabolic activity and interspecific competition, driving spatiotemporal heterogeneity in community structure [12]. For instance, summer heatwaves often coincide with blooms of *Cyanobacteria* [13,14], while winter cooling enhances the organic matter decomposition capacity of heterotrophic bacteria [15]. Deciphering these environment–microbe interactions is essential for predicting water quality trends and formulating ecological restoration strategies.

In recent years, the water quality stability of long-distance water diversion projects has attracted widespread attention [16,17]. The Middle Route of the South-to-North Water Diversion Project is the world’s largest water resource allocation project spanning different basins and climate zones. As an important inter-basin water diversion project, it aims to alleviate water scarcity in northern China and ensure ecological environment and water quality security along the route [16,18]. Although existing studies have gradually focused on related fields of artificial water diversion projects, there are still obvious limitations: First, most existing studies focus on natural rivers or lake systems, and research on artificial channels, a special ecosystem, is relatively scarce. There is a lack of systematic explanation of the dynamic coupling relationship between water quality and microbial communities in large-scale artificial water conveyance corridors such as the Middle Route main canal. Second, although studies by Liu et al. [19] on the Eastern Route of the South-to-North Water Diversion Project have revealed the characteristics of bacterial community changes during the water conveyance period, Zhang et al. [20] pointed out that the dynamics of microbial groups in the Middle Route are driven by seasonality, and Huang et al. [21] explored the assembly mechanism of biofilm microbial communities in the Middle Route, these studies either are limited to a single project section or focus on community assembly processes. There is no systematic understanding of the seasonal response mechanisms between microbial communities and key water quality factors across the entire scale of the Middle Route project, especially the lack of in-depth analysis of the synergistic effects of environmental factors on microbial community structure in different seasons. Third, few existing studies have explored the dynamic changes of the interaction network between microbial communities themselves and with environmental factors in MRP on a seasonal scale, nor their impact on ecosystem stability, making it difficult to reveal the adaptive strategies of microbial communities under low or high temperature stress and their ecological functional significance. Given the growing concerns over water quality degradation, understanding the dynamics of aquatic microbial communities and their interactions with environmental factors has emerged as a critical focus in aquatic ecosystem research. This study selected the Middle Route of the South-to-North Water Diversion Project—a mega-engineered waterway—as the study area. By analyzing seasonal (summer and winter) monitoring data on water quality and microbial communities, we aimed to address two key questions: 1. To uncover the composition, spatial distribution, and diversity patterns of microbial communities in the SNWD-MRP. 2. To elucidate how the microbial community structure responds to environmental drivers, including water temperature and nutrient availability.

## 2. Materials and Methods

### 2.1. Study Area and Sampling

**Figure 1:**
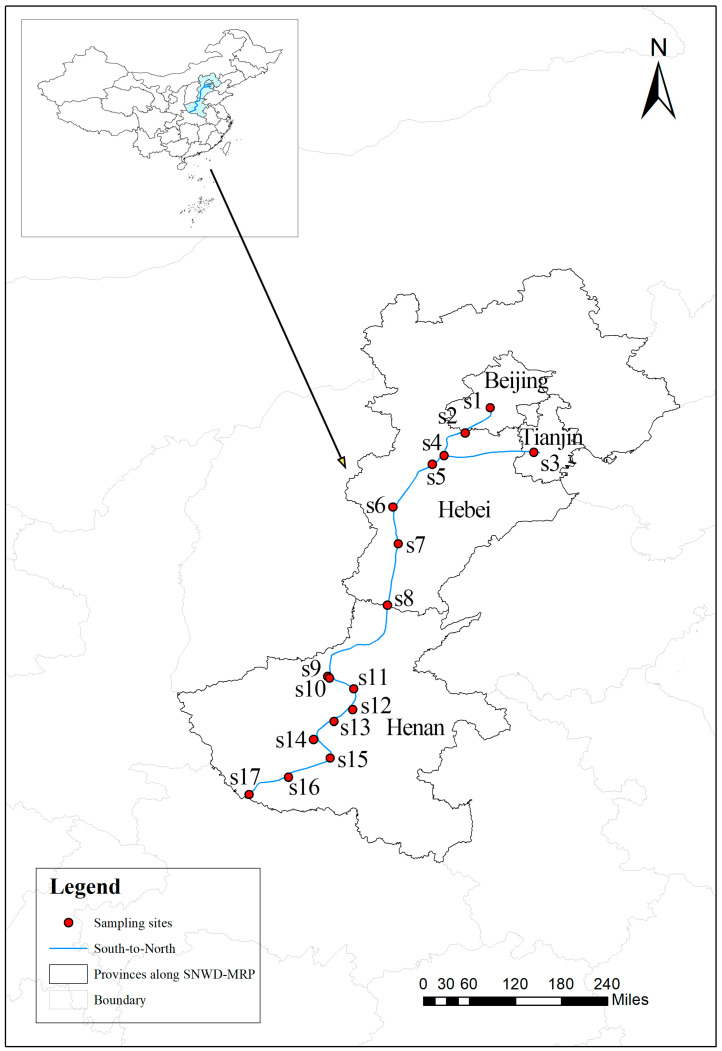
Map of the study area and sampling site layout.

The study area encompassed the main channel of the SNWD-MRP. Based on hydrological monitoring data, the study period was divided into summer and winter. Seventeen sampling sites (labeled S1–S17, Figure 1) were established along the channel from north to south: S1–S2 (Beijing), S3–S4 (Tianjin), S5–S8 (Hebei Province), S9–S14 (Henan Province), and S15–S17 (canal headwater region) (Figure 1).

Water quality parameters were measured at each site. Surface water samples (1.5 L) were collected at 0.5 m depth using sterilized samplers (Shengli Chemical Technology Co., Ltd., Nanjing, China), stored at 4 °C, and transported to the laboratory within 24 h for analysis. Water quality physicochemical parameters such as chemical oxygen demand by potassium permanganate (COD_Mn_), chemical oxygen demand by potassium dichromate (COD), 5-day biochemical oxygen demand (BOD_5_), ammonium nitrogen (AN), total phosphorus (TP), total nitrogen (TN), were determined following APHA standard methods [22]. The other conventional water parameters, including dissolved oxygen (DO), water temperature (WT), and pH, were measured in situ by a portable multi-parameter water quality analyzer (Xylem Inc., Washington, DC, USA). For microbial analysis, water samples were vacuum-filtered through 0.2 μm polycarbonate membranes (Whatman, Marlborough, MA, USA). Prior to filtration, the filtration apparatus was disinfected with sodium hypochlorite and rinsed thoroughly. Filter membranes were flash-frozen in liquid nitrogen and transported to the laboratory. All microbial sample analyses were performed in triplicate for each sampling site to ensure reproducibility.

### 2.2. DNA Extraction, Sequencing, and Processing

Genomic DNA was extracted from filter membranes using the sodium dodecyl sulfate (SDS) method. DNA concentration and purity were assessed via 1% agarose gel electrophoresis, and samples were diluted to 1 ng/μL. The V3–V4 hypervariable regions of the bacterial 16S rRNA gene were amplified using primers 338F (5′-ACTCCTACGGGAGGCAGCAG-3′) and 806R (5′-GGACTACHVGGGTWTCTAAT-3′). Each 50 μL PCR reaction contained 15 μL Phusion^®^ High-Fidelity PCR Master Mix (New England Biolabs, Ipswich, MA, USA), 0.2 μM primers, and 10 ng DNA. Thermocycling conditions included initial denaturation at 98 °C for 1 min, followed by 30 cycles of 98 °C for 10 s, 50 °C for 30 s, and 72 °C for 30 s, with a final extension at 72 °C for 5 min. PCR products were electrophoresed on 2% agarose gels, purified using the Qiagen Gel Extraction Kit (Qiagen, Hilden, Germany), and pooled equimolarly. Sequencing libraries were prepared with the NEBNext^®^ Ultra™ II DNA Library Prep Kit (New England Biolabs, Ipswich, MA, USA) (Cat. No. E7645) and quantified using a Qubit^®^ 2.0 Fluorometer (Thermo Fisher Scientific, Waltham, MA, USA) and Agilent Bioanalyzer 2100 (Agilent Technologies Inc., Santa Clara, CA, USA). Paired-end sequencing (250 bp) was performed on the Illumina NovaSeq platform (Illumina Inc., San Diego, CA, USA).

### 2.3. Bioinformatics Processing

Raw reads were demultiplexed based on unique barcodes, and primer sequences were trimmed. FLASH v1.2.11 was used to merge paired-end reads with overlapping regions. Quality filtering of raw tags was conducted using fastp v0.20.0 to obtain clean tags. Chimeras were removed via the USEARCH v11.0 pipeline, and operational taxonomic units (OTUs) were clustered at 97% similarity. Representative OTU sequences were taxonomically annotated using the RDP classifier Bayesian algorithm in QIIME with the SILVA database (v138). The OTU abundance data and diversity indices reported in the results were derived from the average values of these triplicate analyses.

### 2.4. Statistical Analysis

RDA and Mantel tests were performed using the “vegan” package in R v4.3.1 to assess relationships between microbial communities and environmental factors. In RDA analysis, the envfit function is used to conduct significance tests on variables. Ecological networks were constructed with the igraph package and visualized using Gephi v0.9.2. Network parameters (e.g., node connectivity, edge density) were calculated to evaluate microbial interaction complexity.

## 3. Results

### 3.1. Spatiotemporal Variations in Water Quality Parameters Along the SNWD-MRP

**Figure 2:**
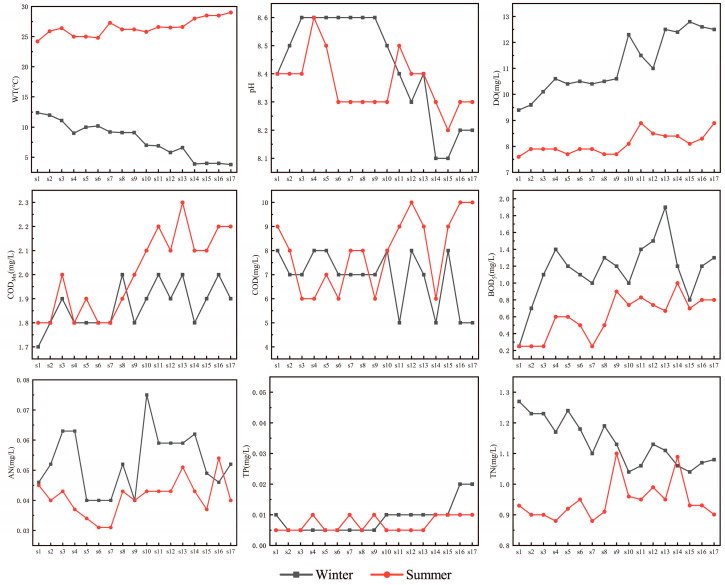
Spatiotemporal variations in water quality parameters along the Middle Route of the South-to-North Water Diversion Project.

As illustrated in Figure 2, significant spatiotemporal differences in water quality parameters were observed across 17 sampling sites along the SNWD-MRP during summer and winter. Seasonal variations in water temperature were most pronounced, with summer WT ranging from 24.2 to 29.0 °C (mean: 26.5 ± 1.4 °C) and winter WT from 3.8 to 12.4 °C (mean: 7.9 ± 2.9 °C). DO levels were significantly higher in winter (mean: 11.2 ± 1.1 mg/L) than in summer (mean: 8.1 ± 0.4 mg/L), while pH remained stable (8.1 ± 0.1–8.6 ± 0.2) across seasons.

COD_Mn_ showed minimal seasonal variation, with summer and winter means of 2.0 ± 0.2 mg/L and 1.9 ± 0.1 mg/L, respectively. In contrast, COD was significantly higher in summer (7.9 ± 1.5 mg/L) than in winter (6.9 ± 1.2 mg/L). Both BOD_5_ and AN showed higher values in winter than in summer.

Nutrient dynamics were complex: TP concentrations were consistently low (0.005–0.02 mg/L), with slightly higher winter averages (0.009 ± 0.005 mg/L vs. 0.007 ± 0.003 mg/L in summer). TN ranged from 0.88 to 1.27 mg/L, peaking in winter (mean: 1.14 ± 0.1 mg/L vs. 0.95 ± 0.1 mg/L in summer). According to China’s National Surface Water Quality Standards (GB3838-2002) [23], water quality at all sites met Class I standards in winter, while COD_Mn_ levels at northern sites (S1–S8) marginally exceeded Class I thresholds during summer.

### 3.2. Microbial Community Composition Along the SNWD-MRP

**Figure 3:**
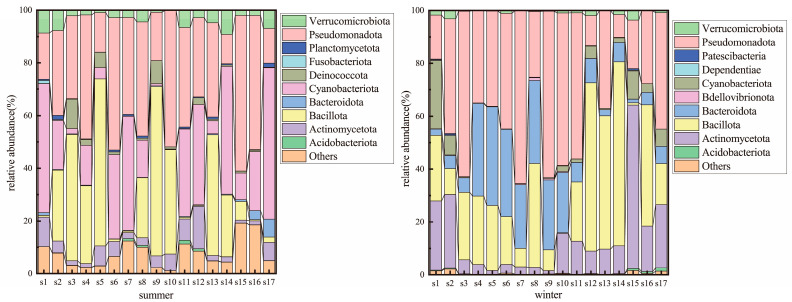
Microbial community composition along the Middle Route of the South-to-North Water Diversion Project during summer and winter.

Based on 16S rRNA gene sequencing data, bacterial communities exhibited significant spatial heterogeneity at the phylum level (Figure 3). In winter, *Pseudomonadota* (38.24%), *Bacillota* (26.57%), *Bacteroidota* (15.39%), and *Actinomycetota* (13.76%) dominated, collectively accounting for 93.96% of sequences. *Pseudomonadota* was ubiquitously abundant (8.66–65.47%), peaking at S7 (65.47%) and S9 (62.92%). *Bacillota* reached its maximum abundance (69.60%) at S14, while *Cyanobacteria* showed high variability (0.07–25.79%), peaking at S1 (25.79%).

In summer, *Pseudomonadota* (34.21%), *Cyanobacteria* (23.16%), and *Bacillota* (22.12%) emerged as core phyla. Notably, *Cyanobacteria* abundance surged from 3.83% in winter to 23.16% in summer, becoming the second most dominant phylum. Its abundance was highest at S1 (48.95%), S14 (48.51%), and S17 (57.36%). Other phyla, including unclassified groups (7.67%), *Actinomycetota* (5.04%), *Verrucomicrobiota* (3.92%), and *Deinococcota* (2.13%), showed more uniform distributions.

### 3.3. Microbial Diversity Patterns

**Figure 4:**
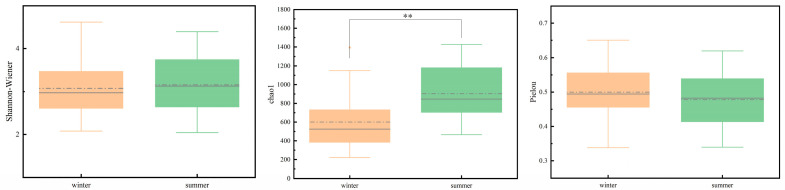
Diversity patterns of microbial communities along the SNWD-MRP. ** indicates *p* < 0.01.

Seasonal shifts in microbial diversity indices were evident (Figure 4). The Shannon–Wiener index showed minor differences between winter (2.08–4.62, mean: 3.08) and summer (2.04–4.39, mean: 3.16). In contrast, the Chao1 index was significantly higher in summer (mean: 903.23, range: 466.04–1427.50) than in winter (mean: 601.22, range: 222.64–1393.93) (*p* < 0.01). Pielou’s evenness index was marginally higher in winter (0.34–0.65, mean: 0.50) compared to summer (0.34–0.62, mean: 0.48), though seasonal differences were negligible.

### 3.4. Impacts of Water Quality Factors on Microbial Communities

**Figure 5:**
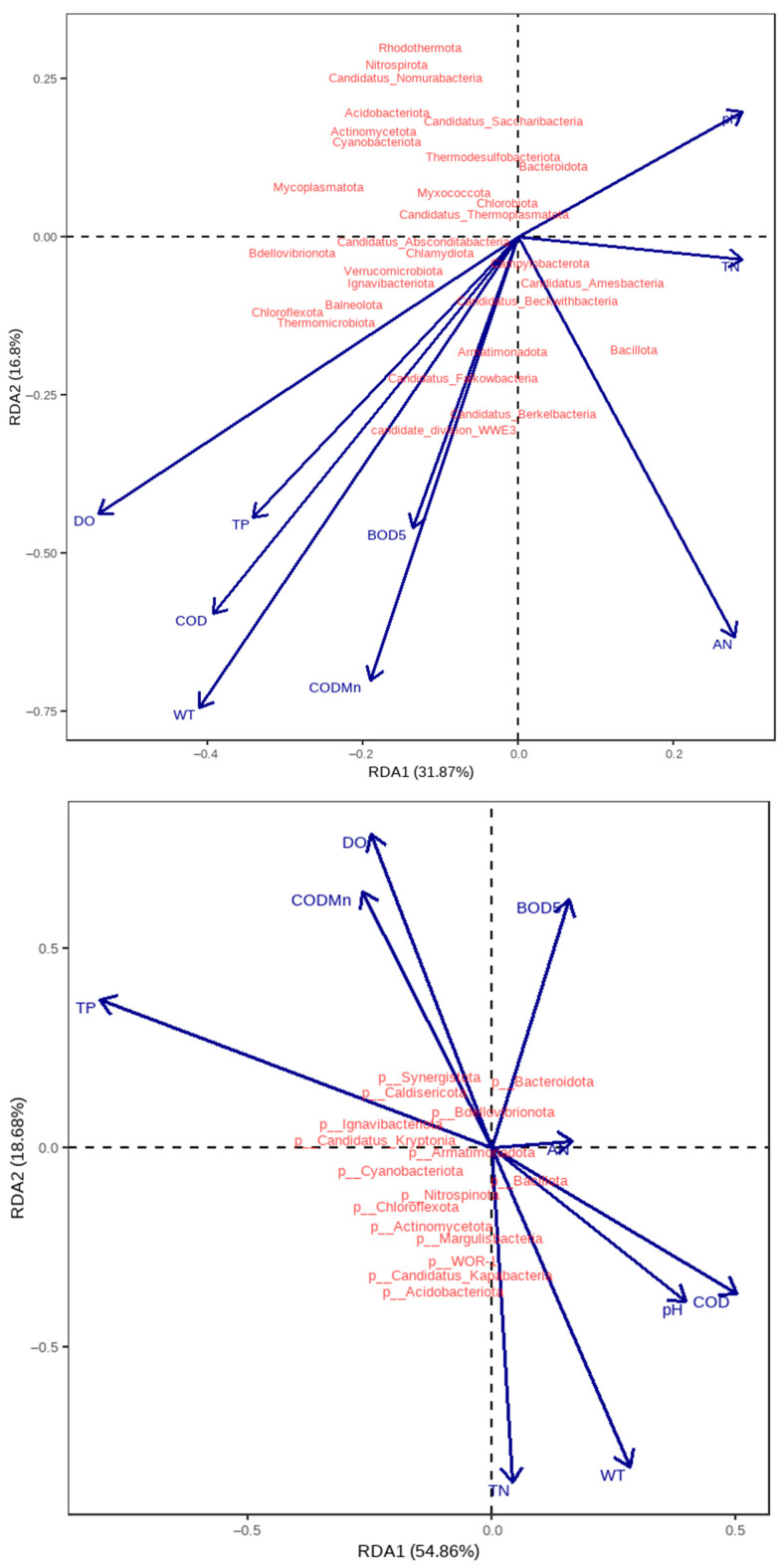
RDA ordination of microbial communities and environmental drivers. Upper: summer. Lower: winter.

RDA was employed to identify key environmental drivers of bacterial community structure along the MR-SNWDP (Figure 5). In summer, the variables that significantly affect community composition include DO, COD_Mn_, COD, and AN, with the first two axes (RDA1 and RDA2) explaining 49.88% of the total constrained variance. In winter, COD_Mn_, TP, and TN significantly influenced bacterial communities, with RDA1 and RDA2 accounting for 78.93% of the variance, indicating stronger explanatory power of environmental factors during colder months. Notably, COD_Mn_ emerged as a critical driver across both seasons.

### 3.5. Microbial Community-Environment Interactions

**Figure 6:**
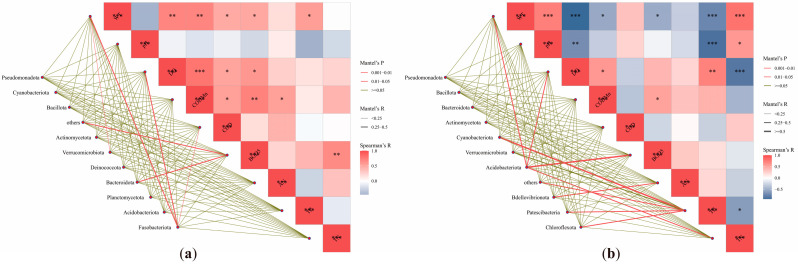
Mantel test between water quality parameters and bacterial communities in the SNWD-MRP (**a**): summer; (**b**): winter. * indicates *p* < 0.05, ** indicates *p* < 0.01, and *** indicates *p* < 0.001.

Mantel tests between the top 10 bacterial phyla and physicochemical factors revealed seasonally divergent correlations (Figure 6a,b). In summer, unclassified phyla and *Bacteroidota* exhibited strong associations with BOD_5_, while *Fusobacteriota* was closely linked to WT, DO, and COD. *Verrucomicrobiota* showed a notable relationship with WT. Spearman correlation analysis indicated significant positive correlations among WT, DO, COD_Mn_, COD, BOD_5_, and TP, with DO positively correlated to COD_Mn_, COD, and BOD_5_. COD_Mn_ was positively associated with COD, BOD_5_, and AN, while BOD_5_ positively correlated with TN.

In winter, Cyanobacteria displayed a significant positive correlation with TP, whereas Acidobacteriota was associated with WT, DO, TN, and BOD_5_. Unclassified phyla, Patescibacteria, and Chloroflexota were closely related to TP, and Bdellovibrionota correlated with AN. Spearman analysis revealed contrasting patterns: WT positively correlated with pH and TN but negatively with DO, COD_Mn_, BOD_5_, and TP. pH showed negative correlations with DO and TP but a positive correlation with TN. DO was positively correlated with COD_Mn_ and TP but negatively with TN, while COD_Mn_ and BOD_5_ were positively linked. TP and TN exhibited a significant negative correlation.

### 3.6. Seasonal Characteristics of Ecological Networks Between Water Quality and Microbial Communities

**Figure 7:**
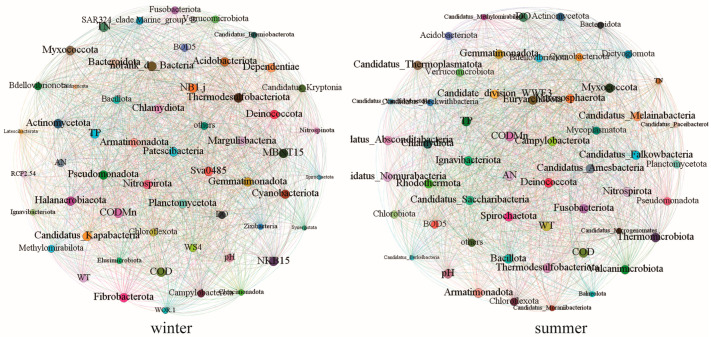
Ecological network between water quality parameters and microbial communities.

Ecological networks integrating physicochemical factors and microbial taxa were constructed to assess seasonal variations in network structure and function (Figure 7, Table 1). The summer network comprised 55 nodes and 1464 edges (average degree: 53.24), whereas the winter network expanded to 56 nodes and 1523 edges (average degree: 54.39), indicating enhanced complexity and connectivity of microbial-environment interactions during colder months. Both node and edge connectivity increased from 50 (summer) to 52 (winter). The near-maximal graph density (>0.98) and clustering coefficient (>0.98) observed in both seasons suggested ultra-dense network structures, with winter’s higher connectivity further enhancing network stability.

Strong correlations between TP and *Nitrospirota* and between TN and *Myxococcota* in the winter network implied nutrient-driven regulation of nitrifying and predatory bacterial populations. The increased edge number and connectivity in winter may stem from intensified microbial interactions (e.g., cometabolism or stress response) under low-temperature conditions. Additionally, the reduced average path length (0.005 in winter vs. 0.011 in summer) indicated improved metabolic efficiency, likely attributable to stabilized microenvironments resulting from diminished hydraulic disturbances during winter.

## 4. Discussion

### 4.1. Temporal and Spatial Differences in Microbial Communities

This study found that there is significant spatial heterogeneity in the microbial communities among different sampling sites in the main channel of SNWD-MRP. For example, *Cyanobacteriota* was significantly enriched (relative abundance > 50%) at southern sites such as S17 and S14 in summer. This difference is closely related to the local characteristics of water quality parameters along the route and regional geographical features. In summer, the COD_Mn_ and TN concentration at southern sites (S10–S17) were higher than those at northern sites, which may have promoted the proliferation of nutrient-dependent *Cyanobacteriota*, reflecting the impact of local environmental filtering on microbial community composition in artificial water diversion projects. Compared with regional and similar studies, the spatial heterogeneity characteristics in this study differ from those reported in studies on the Eastern Route of the South-to-North Water Diversion Project. In the Eastern Route, the spatial heterogeneity of bacterial communities significantly decreased during the water transfer period [19]. In contrast, the Middle Route exhibits more prominent spatial heterogeneity due to its wider span across climate zones, more complex terrain, and being a closed channel. This is consistent with the study by Huang et al. on biofilm microbiota in the Middle Route, confirming the general pattern that local environmental heterogeneity drives microbial spatial differentiation [21]. Meanwhile, this study found that aquatic microbiota is closely associated with the spatial distribution of water nutrients. Compared with natural rivers, the spatial differences in their microorganisms are mainly driven by watershed topography, land use intensity, and environmental stress caused by human activities [24,25,26,27], the Middle Route, as an artificially regulated system, has spatial heterogeneity that is more dependent on the local water environment, reflecting the differences between artificial and natural systems.

The microbial communities in the SNWD-MRP exhibited pronounced seasonal succession, with core dominant phyla closely linked to fluctuations in water temperature (WT), dissolved oxygen (DO), and nutrient availability. *Pseudomonadota*, *Bacillota*, *Bacteroidota*, *Cyanobacteria*, and *Actinomycetota* dominated the bacterial communities, all of which are commonly reported in inland rivers [20]. WT, a key seasonal driver, directly or indirectly modulates community composition by selecting for temperature-adapted taxa [3,28]. In this study, high summer temperatures (24.2~29.0 °C) significantly promoted the proliferation of the Cyanobacteria, with its relative abundance surging from 3.83% in winter to 23.16%, and forming accumulations at sites S1, S14, and S17 (Figure 3). This result is consistent with the global-scale study by Kosten et al. [15], which showed that increased water temperature is significantly positively correlated with cyanobacterial biomass. Moreover, it synergistically enhanced photoautotrophic processes together with local summer nutrient inputs (such as the peak TN at S1 and elevated TP at S14/S17) [29]. Notably, similar seasonal cyanobacterial blooms have also been observed in the Eastern Route of the South-to-North Water Diversion Project. Liu et al. [19] found a correlation between high temperatures during water conveyance periods and algal growth. However, due to stronger hydrodynamic disturbances in the Eastern Route (with larger variations in water transfer flow rates), the degree of cyanobacterial accumulation is lower than in the study area, suggesting that the role of water temperature may be regulated by hydrodynamic conditions.

*Pseudomonadota*, the most abundant phylum, showed higher winter dominance, potentially due to low-temperature acclimation. Previous studies indicate that Pseudomonas (a genus within *Pseudomonadota*) thrives in cold-adapted biofilms at 5 °C [30]. *Bacteroidota* declined in summer (15.39% in winter vs. lower summer abundance), likely suppressed by Cyanobacteria blooms. *Bacillota*’s winter prevalence (26.57%) may reflect its adaptation to nutrient-rich conditions (winter TN/TP > summer) [29], while *Actinomycetota*’s seasonal increase aligns with elevated DO levels favoring aerobic taxa [31,32].

### 4.2. Water Quality-Microbial Community Interactions

Water quality in aquatic systems is governed by physicochemical-microbial interplay. Integrating these parameters elucidates how bacteria utilize specific nutrients [33]. Redundancy analysis (RDA) identified COD_Mn_ as a key driver of bacterial structure across seasons, underscoring organic pollutants as critical regulators of microbial growth [34]. The stronger explanatory power of winter RDA models (78.93% variance explained) highlights the heightened influence of TP and TN under cold conditions. Mantel tests confirmed tight associations between DO and multiple phyla, as well as Cyanobacteria-TP correlations, consistent with nutrient-driven blooms at high-TP/TN sites. The higher Chao1 diversity index in summer (summer: 903.23 vs. winter: 601.22) may be attributed to elevated temperatures. On one hand, WT directly affects the activity of microbial metabolic enzymes; increased water temperature promotes the metabolism and reproduction of such bacteria. On the other hand, water temperature acts by indirectly regulating environmental factors: high temperatures promote the growth of phytoplankton, accelerate CO_2_ consumption and O_2_ release, alter water pH and dissolved oxygen concentrations, and phytoplankton absorption of inorganic nutrients changes the availability of nutrients in the water. These environmental changes further affect bacterial community structure, facilitating the proliferation of bacteria adapted to the new environment. Finally, as water temperature rises, the phylogenetic diversity of planktonic bacteria increases, and the number of dominant species grows. Higher phylogenetic diversity implies more complex interspecific relationships and niche differentiation, enabling more bacterial species to coexist in the warmed environment, thereby enhancing community richness [35].

### 4.3. Seasonal Drivers of Water Quality–Microbial Networks

Ecological network analysis revealed intensified microbial–environment interactions in winter (Table 1, Figure 7). The winter network exhibited higher complexity (1523 edges vs. 1464 in summer), connectivity (54.39 vs. 53.24 average degree), and stability (node/edge connectivity: 52 vs. 50), despite near-maximal graph density (>0.98) and clustering coefficients (>0.98) in both seasons. Water quality analysis showed that the concentrations of DO, BOD_5_, TN, and AN at almost all sites were higher in winter than in summer (Figure 2). Suitable nutrient levels can enhance the complexity of microbial networks, mainly because moderate nutrient stress eliminates species sensitive to nutrient changes through environmental filtering, while the remaining microbial taxa are more likely to form tightly connected community structures due to niche conservatism. Meanwhile, intensified resource competition drives microorganisms to develop more complex cooperative or competitive relationships—for instance, some taxa utilize limited nutrients through synergistic metabolism, while others occupy specific niches through competition. These interactions increase the connectivity (e.g., higher average degree) and clustering degree (e.g., higher average clustering coefficient) of the network, thereby making the network module structure more complex [36].

Notably, the TN concentration in this study ranges from 0.88 to 1.27 mg/L, which falls within the suitable nutrient range that supports microbial reproduction [37], providing the fundamental nitrogen conditions for the formation of the aforementioned network complexity. Furthermore, the strong correlation between TN and *Myxococcota* is a concrete manifestation of microbial cooperative relationships under such suitable nutrient levels—through synergy in nutrient-mediated nitrogen cycling, they further strengthen interspecific metabolic associations, acting as key drivers for the enhancement of network connectivity [38,39]. In addition, this TN concentration does not reach the threshold for triggering massive proliferation of Cyanobacteria (>0.15 mg/L) [40,41]. It not only maintains the balance of nitrogen supply but also avoids excessive niche occupation by a single taxon that would disrupt the complex structure of the network, which also indirectly corroborates the supporting role of moderate nutrient levels in network stability.

## 5. Conclusions

This study systematically elucidated the spatiotemporal dynamics of microbial communities and their interactions with water quality parameters in the SNWD-MRP, providing critical insights into the ecological mechanisms governing engineered water systems. Key findings include the following:

(1) Microbial communities exhibited distinct seasonal patterns, driven by temperature, DO, and nutrient fluctuations. The summer proliferation of Cyanobacteria was linked to elevated temperatures and nutrient inputs (TN/TP), while winter dominance of Pseudomonadota and Bacillota reflected adaptations to cold conditions and organic matter degradation.

(2) RDA analysis revealed that the COD_Mn_ served as a pivotal driver shaping microbial communities across both summer and winter, while TP and TN exerted stronger influences during winter. Mantel tests further validated these findings, demonstrating significant correlations between DO and multiple bacterial phyla, as well as a robust association between Cyanobacteria and TP. These results collectively highlight the synergistic regulation of microbial functional differentiation by dissolved oxygen and nutrient availability.

(3) Winter conditions fostered more complex and stable microbial networks compared to summer, driven by moderate nutrient levels that optimized environmental filtering, niche conservatism, and interspecific interactions

## Figures and Tables

**Table 1:** Characteristic parameters of ecological networks.

Parameter	Summer	Winter
nodes number	55	56
edges number	1464	1523
average degree	53.24	54.39
nodes connectivity	50	52
edges connectivity	50	52
average path length	0.011	0.005
graph diameter	0.044	0.048
graph density	0.986	0.989
clustering coefficient	0.986	0.989
betweenness centralization	2.26 × 10^−5^	1.23 × 10^−5^
degree_centralization	0.014	0.011

## Data Availability

The data presented in this study are available on request from the corresponding author due to the data privacy requirements of the South-to-North Water Diversion Project.
